# Hypoalgesia Induced by Reward Devaluation in Rats

**DOI:** 10.1371/journal.pone.0164331

**Published:** 2016-10-20

**Authors:** Ana María Jiménez-García, Leandro Ruíz-Leyva, Cruz Miguel Cendán, Carmen Torres, Mauricio R. Papini, Ignacio Morón

**Affiliations:** 1 Department of Pharmacology, Biomedical Research Center (CIBM) and Institute of Neuroscience, Faculty of Medicine, University of Granada, Campus Ciencias de la Salud, 18016, Granada, Spain; 2 Department of Psychology, University of Jaén, Campus Las Lagunillas, 23071, Jaén, Spain; 3 Department of Psychology, Texas Christian University, Fort Worth, TX, 76129, United States of America; 4 Department of Psychobiology and Research Center for Mind, Brain, and Behavior (CIMCYC), University of Granada, Faculty of Psychology, Campus Cartuja, 18071, Granada, Spain; University of Florida, UNITED STATES

## Abstract

Reduced sensitivity to physical pain (hypoalgesia) has been reported after events involving reward devaluation. Reward devaluation was implemented in a consummatory successive negative contrast (cSNC) task. Food-deprived Wistar rats had access to 32% sucrose during 16 sessions followed by access to 4% sucrose during 3 additional sessions. An unshifted control group had access to 4% sucrose throughout the 19 sessions. Pain sensitivity was measured using von Frey filaments (Experiment 1) and Hargreaves thermal stimuli (Experiment 2) in pretraining baseline, 5 min, and 300 min after either the first (session 17) or second (session 18) devaluation session in the cSNC situation. Sucrose consumption was lower in downshifted groups relative to unshifted groups during postshift sessions—the cSNC effect. Hypoalgesia was observed in downshifted groups relative to unshifted controls when pain sensitivity was assessed 5 min after either the first or second devaluation session, regardless of the pain sensitivity test used. Both pain sensitivity tests yielded evidence of hypoalgesia 300 min after the second downshift session, but not 300 min after the first devaluation session. Whereas hypoalgesia was previously shown only after the second devaluation session, here we report evidence of hypoalgesia after both the first and second devaluation sessions using mechanical and thermal nociceptive stimuli. Moreover, the hypoalgesia observed 300 min after the second devaluation session in both experiments provides unique evidence of the effects of reward loss on sensitivity to physical pain 5 hours after the loss episode. The underlying neurobehavioral mechanisms remain to be identified.

## Introduction

Sensitivity to physical pain is influenced by a variety of emotional states [1,2], including stress induced by immobilization [3] and food deprivation [4]. The emotional modulation of physical pain also occurs after an experience involving the devaluation of a large reward, as documented below. In the present experiments, reward devaluation was implemented in terms of the consummatory successive negative contrast (cSNC) task [5]. In a typical cSNC experiment, animals receive free access for 5 min to a high-value sucrose solution (typically 32% sucrose) during several daily sessions, followed by several sessions of access to 4% sucrose. Performance during these downshift sessions is compared to the consummatory behavior of animals that have always received access to 4% sucrose (unshifted controls). The cSNC effect involves a suppression of consummatory behavior in 32-to-4% sucrose animals followed by a recovery of levels similar to those of unshifted controls. The initial suppression (typically observed during the first devaluation session) and the recovery that follows (typically starting during the second devaluation session) are dissociable stages of the cSNC effect, as extensively demonstrated by Flaherty’s research on cSNC [5]. Several sources of evidence suggest that cSNC modulates and is also modulated by physical pain. In one experiment [6], sensitivity to physical pain was assessed in terms of the paw-withdrawal latency in the hot plate after reward devaluation. The results showed reduced pain sensitivity (i.e., increased paw-withdrawal latency) after the second 32-to-4% sucrose downshift session, but not after the first one, relative to unshifted controls. Conversely, a subcutaneous formalin injection in a hind paw before the first and second downshift sessions enhanced the consummatory suppression induced by either a 32-to-4% or a 16-to-4% sucrose downshift [7]. These studies add to growing evidence suggesting that situations that actually or potentially involve tissue damage share common underlying mechanisms with situations involving reward devaluation. Based on extensive evidence, Papini et al. [8] suggested a connection between physical pain (tissue damage) and psychological pain (reward loss) that invites further comparisons between these two sets of phenomena. For example, opioid ligands known to modulate physical pain also modulate cSNC, either during the first downshift session ([D-Pen2,D-Pen5]enkephalin, a selective delta-receptor agonist; [9]), during the second downshift session (U50,488H, a selective kappa-receptor agonist; [10]), or during both sessions (morphine; [11]). Two opioid-receptor antagonists enhance the cSNC effect either selectively during the first downshift session (naltrindole, a selective delta receptor antagonist) or during both the first and second downshift sessions (naloxone, a nonselective opioid receptor antagonist) [12]. Similarly, lesions of brain areas involved in pain processing, such as the anterior cingulate cortex, also affect recovery from reward downshift from the second downshift session onward [13].

The goal of these experiments was to look for evidence of the modulation of physical pain by reward devaluation during the first and second downshift sessions by using different techniques to assess pain sensitivity: the von Frey test and the Hargreaves test. These tests estimate pain thresholds in terms of paw-withdrawal latency applying a localized mechanical or thermal stimulus. Both are relatively easy to apply, and have been extensively used with rodents [14,15,16,17] and even humans [18]. Additionally, these experiments were designed to provide evidence of the postsession time course of the effect by measuring pain sensitivity 5 and 300 min after the end of the reward devaluation session. The von Frey test consists of filaments varying in thickness that are pressed against the skin of a hind paw. Detection of the mechanical pressure results in paw withdrawal, thus providing an objective measure of mechanical hypo- or hyperalgesia. The Hargreaves test estimates pain sensitivity also in terms of paw withdrawal, but after applying a localized thermal stimulus [16]. These techniques differ in the type of nociceptive stimulus used—mechanical or thermal. Moreover, these techniques stimulate only a small patch of skin in a hind leg and, therefore, are probably more sensitive than other techniques, such as the hot plate test previously used in a similar experiment [6]. An additional advantage of the von Frey and Hargreaves tests for situations involving repeated testing is that there is little evidence that they induce aversive conditioning in intact animals [19]. By contrast, just a single exposure using the hot plate test has been shown to induce aversive conditioning [20]. Thus, using the von Frey test (Experiment 1) and Hargreaves test (Experiment 2) would tend to minimize a possible interaction between reward devaluation and aversive conditioning. Based on previous results [6], a hypoalgesic effect was expected after the second downshift session, but not after the first downshift session, at least when testing occurred 5 min after the session. Testing 300 min after both sessions was expected to yield little or no evidence of changes in pain sensitivity. While there is no available evidence describing the postsession time course of pain sensitivity effects after reward devaluation, posttraining drug manipulations in the cSNC task typically failed to show effects after a 180-min interval [21,22].

## Materials and Method

### Subjects

In Experiment 1, the subjects were 41 male Wistar rats purchased from Harlan Laboratories (Barcelona, Spain). In Experiment 2, the subjects were 40 male Wistar rats purchased from Charles Rivers (Les Oncins, France). A parvovirus contamination in rats from Harlan Laboratories explains the switch of vendors; however, quarantine (one week) and habituation to the laboratory were done as usual. Rats were approximately 75 days old at the beginning of each experiment. The mean (±SEM) ad lib weight of all the rats was 281.2 (±2.1) g. Rats were housed individually in polycarbonate cages with ad lib water, in a room with constant temperature (24°C) and humidity (50–60%). Animals were housed under a 12:12 h cycle of light: darkness (lights on at 08:00 h) and food deprived to 82–85% of their ad lib weights throughout the experiment. Deprivation levels were maintained by providing rat chow at least 5–50 min after the end of all behavioral testing. In Experiment 1, rat chow was from Harlan, Mucedola, Italy; in Experiment 2, rat chow from Envigo (formerly Harlan), Barcelona, Spain. Water was continuously available in the home cage (these rats were never water deprived). These animals participated in a previous experiment in an instrumental successive negative contrast (iSNC) task in a runway situation, with solid food pellets as reward. These animals also had postsession access to either 2% ethanol or water. Assignment to the new conditions was matched as far as possible for prior experience. In Experiment 1, the numbers of rats with downshifted/unshifted prior experience were 6/4, 4/6, 6/5, and 6/4, respectively, for Groups 32/17, 4/17, 32/18, and 4/18. In Experiment 2, the equivalent numbers were 6/4, 6/4, 4/6, and 4/6, respectively, for Groups 32/17, 4/17, 32/18, and 4/18. As for prior ethanol/water exposure, the numbers for the same groups were 7/3, 7/3, 8/3, and 8/2 in Experiment 1, and 5/5, 5/5, 5/5, and 5/5 in Experiment 2. The experimental protocols were approved by the University of Granada Research Ethics Committee.

### Apparatus

All the behavioral procedures (cSNC and pain sensitivity measures) were conducted in the same lab room. The cSNC procedure was identical for both experiments. cSNC training was carried out in four boxes made of clear Plexiglas and measuring 30 x 30 x 15 cm (L x H x D). A graduated cylinder (in 0.01-ml units) containing the sucrose solution was introduced to the inside in the center of one of the lateral walls of the box. The amount of sucrose solution consumed by each animal in each session (in milliliters) was obtained by subtracting the amount of sucrose solution recorded after the session from the amount provided before the session. Animals licked the solutions from a metallic sipper tube protruding 3 cm inside the box. Sucrose solutions were prepared weight/weight by mixing 32 g of commercial sugar for every 68 g of distilled water (32% sucrose) and 4 g of sugar for every 96 g of distilled water (4% sucrose).

The von Frey and Hargreaves tests were conducted in two boxes, also made of clear Plexiglas, and measuring 20 x 20 x 24 cm (L x H x D). For the von Frey test, the floor was made of aluminum bars. For the Hargreaves test the floor was made of glass. Pain sensitivity was determined by measuring the paw-withdrawal response to a punctate mechanical or thermal stimulation of one of the hind paws. In the von Frey test, stimulation involved applying one of a range of 9 von Frey filaments (Touch-Test Sensory Evaluators, North Coast Medical, CA, USA) ranging from 0.4 to 10 g (3.92–98.1 mN) to a hind paw. For the Hargreaves test, radiant heat (42–43°C through the glass floor, for a maximum of 13 s) was applied by a plantar test apparatus (Ugo Basile, Comerio, Italy). Temperature was kept constant throughout the experiment.

### Procedure

To the authors’ knowledge, this is the first experiment exploring the effects of reward downshift in the cSNC situation on physical pain assessed with the von Frey filaments and the Hargreaves thermal test. The protocols implemented here combine testing parameters used separately in prior experiments involving cSNC, von Frey testing, and Hargreaves testing.

cSNC training lasted 19 daily sessions: 16 preshift sessions followed by 3 postshift sessions. Four groups were included: 32/17 (*n* = 10), 4/17 (*n* = 10), 32/18 (*n* = 11 in Experiment 1, *n* = 10 in Experiment 2), and 4/18 (*n* = 10). In group labels, the first number refers to the sucrose concentration administered during preshift sessions (32 or 4% sucrose; all animals had access to 4% sucrose during postshift sessions), whereas the second number refers to the session when the von Frey test or Hargreaves test was administered, either after the first postshift session (17) or the second postshift session (18).

Each day, the animal rack was moved into the experimental room, rats were allowed 15 min in their home cage to settled, and then they were placed in the contrast box. The contrast session lasted 5 min from the first contact with the sipper tube. Session duration for each box was measured manually with digital clocks (Digital Onstart 100). Animals were run in squads of 4 and the order of squads varied across days. At the end of all sessions, every day, contrast boxes were wiped with a wet paper towel and feces were removed when present.

In Experiment 1, Von Frey testing was conducted 5 and 300 min after session 17 for Groups 32/17 and 4/17, and after session 18 for Groups 32/18 and 4/18. Animals received one additional postshift session (19) to determine whether von Frey testing affected consummatory behavior on the following session. To minimize novelty effects with the von Frey boxes, animals were familiarized before the critical test sessions according to the following schedule. Baseline measurements were conducted two days before the start of iSNC training, during a previous phase not reported here (see **Subjects** for a description of previous experience). In addition, rats were exposed to the von Frey boxes for 5 min per session, 5 min after each of eight preshift sessions during the current experiment. During these 8 box-exposure sessions no measurements were taken with von Frey filaments.

In every test, each filament was applied three times for 2–3 s, separated by 5-s intervals using the up-down paradigm [23]. Testing started with the 2-g (19.6 mN) von Frey filament (i.e., the middle of the range). The filament was manually pressed against the paw’s plantar surface with sufficient force to cause a depression in the skin. The paw chosen for stimulation was always the same for a given animal and it was counterbalanced for right and left hind paw within each group (5/5 for three groups and 6/5 for Group 32/18). In each consecutive test, if there was no response to the filament, a stronger stimulus was then selected; if there was a positive response, a weaker one was then used. The response to the filament was considered positive when immediate withdrawal or shaking of the paw was observed. Observers were blind with respect to the contrast assignment of the subject (i.e., 32% vs. 4%).

In Experiment 2, thermal pain sensitivity was assessed with a technique described in [24] with slight modifications. Hargreaves tests were conducted 5 and 300 min after session 17 for Groups 32/17 and 4/17, and after session 18 for Groups 32/18 and 4/18. Animals received one additional postshift session (19) to determine whether Hargreaves testing affected consummatory behavior on the following session. To minimize novelty effects with the Hargreaves test boxes, animals were familiarized before the critical test sessions according to the following schedule. Baseline measurements were conducted two days before the start of iSNC training (see **Subjects**). In addition, rats were exposed to the Hargreaves test boxes for 5 min per session, 5 min after each of eight preshift sessions during Experiment 2. A video camera (Sony Handycam HD) was placed over the Hargreaves test boxes. During these sessions, no video recordings or pain measurements were taken.

Pain sensitivity was assessed for each animal after session 17 in Groups 32/17 and 4/17, or after session 18 in Groups 32/18 and 4/18. The Hargreaves test started with 5 min of habituation to the boxes. During this period, toilet paper was placed on the floor to collect urine and feces. After 5 min, the toilet paper was removed and a beam of radiant heat was focused to the plantar surface of a hind paws with a plantar test apparatus, until the rat made a withdrawal response. Paw withdrawal interrupted the light reflected from the paw onto a photocell and automatically turned off the light and the timer. The latency of the withdrawal response (as an indirect measure of the heat-induced pain threshold) was thus recorded automatically. The intensity of the light was adjusted at the start of Experiment 2 such that average baseline latency was about 13 s. This intensity was never changed. Each rat was tested twice alternately on each hind paw. All the latencies recorded were averaged to obtain a single latency measure per animal. Typically, a measurement was derived from four recordings, two from each hind paw. At least 1 min was allowed between consecutive measurements in the same paw. A cut-off latency time of 30 s was used to avoid skin damage and minimize pain. A Hargreaves test usually lasted 15 min.

### Statistics

In the cSNC situation, the dependent variable was the total amount of sucrose consumed in each session (in milliliters). In Experiment 1, pain sensitivity in the von Frey test was expressed as a mechanical threshold producing a response in 50% of the trials. This paw withdrawal threshold value was calculated using the following formula:

$$50\%\text{ threshold }\left(\text{g}\right)=\;10^{\left(X_{f}\;+\;\kappa \delta \right)}/10,000$$

where X_f_ is the value (in log units) of the final von Frey filament used, κ is the tabular value (see Appendix in [23]) for the pattern of positive/negative responses, and δ is the mean difference (in log units) between stimuli. In Experiment 2, pain sensitivity in the Hargreaves test was expressed in terms of the mean paw-withdrawal latency (in seconds) over measurements taken from each paw. All data analyses were computed with the IBM SPSS Statistics 21 package. The specific analysis of variance used is described in the Results section. Interactions were analyzed with pairwise LSD tests derived from the main analysis. Normality was assessed with the Kolmogorov-Smirnov test whenever a significant effect was detected in downshift sessions and in the von Frey and Hargreaves tests to minimize Type I error. The alpha value was set at the 0.05 level in all statistical analyses.

## Results

### Experiment 1

**Fig 1:**
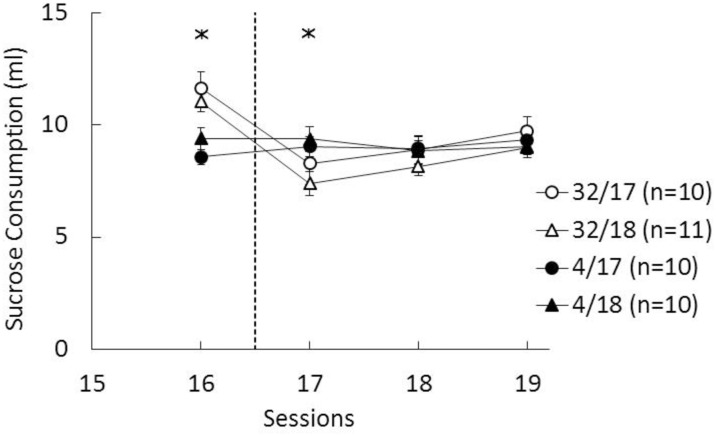
Mean (±SEM) sucrose consumption (ml) during the last preshift session (16) and either session 17 or 18 (Post) depending on the groups. 32: animals exposed to reward devaluation from 32% to 4% sucrose during postshift sessions. 4: animals exposed to an unshifted reward condition, always receiving access to 4% sucrose throughout the experiment. The asterisk reflects a significant difference between both downshifted groups vs. both unshifted controls (see text for details).

A Contrast (32% vs. 4% sucrose) x von Frey (session 17 vs. 18) x Session (1–16) analysis of preshift consummatory performance indicated that 32% groups consumed significantly more sucrose than 4% groups, *F*(1, 37) = 28.65, *p*<0.001, and also there was a significant increase in consumption across sessions, *F*(15, 555) = 87.15, *p*<0.001. Sucrose consumption during the last preshift session (16) and the three postshift sessions (17–19) is presented in Fig 1. Consumption was higher in groups exposed to 32% sucrose than to 4% sucrose on session 16, the last preshift session, *F*(1, 37) = 20.80, *p*<0.001, but there was no difference between groups assigned for von Frey testing on session 17 or 18, or interaction between these two factors, *F*s < 1.91, *p*s>0.17. An analysis of postshift sessions data indicated that there was a weak cSNC effect lasting a single session. This analysis yielded a significant interaction between contrast and postshift session, *F*(2, 74) = 5.68, *p*<0.006, and LSD pairwise comparisons confirmed that downshifted groups consumed significantly less sucrose on session 17 than unshifted controls, *F*(1, 37) = 4.48, *p*<0.05. The differences were not significant for postshift sessions 18 and 19. There was no evidence of deviations from normality on sessions 16–19 (statistics: < 0.18, ps>0.09). Thus, consummatory behavior showed no evidence of a downshift effect on session 18, before the von Frey test was administered in Groups 32/18 and 4/18. Additionally, there was no evidence that von Frey testing in one day affected consummatory behavior in the contrast box the following day.

**Fig 2:**
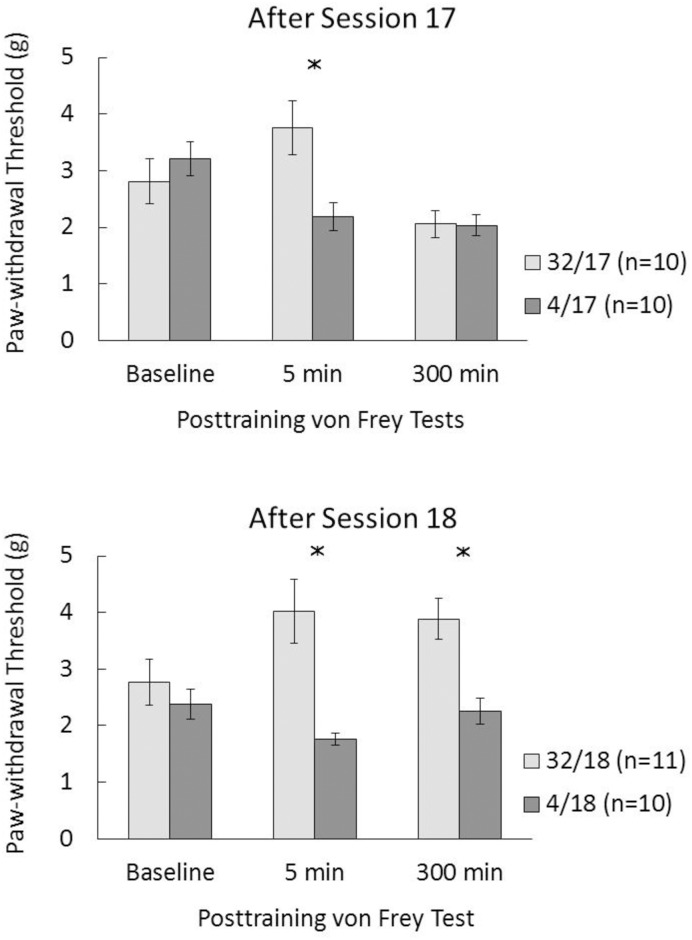
Threshold force (g) for paw withdrawal in the von Frey test during baseline sessions and either 5 or 300 min after sessions 17 (top) or 18 (bottom), depending on the groups. 32: 32-to-4% sucrose downshift. 4: unshifted controls always exposed to 4% sucrose. The asterisks reflect a significant difference between the corresponding downshifted vs. unshifted groups (see text for details).

Fig 2 shows the mean mechanical pain thresholds in groups tested on baseline session, and after session 17 (top) or 18 (bottom). Whereas the baseline measurement was obtained at the same postsession time for all animals, the postshift measurements were obtained after the contrast session. Thus, these three values (baseline, 5 min, and 300 min) are separated by different time interval. Because the relevant comparisons are between downshifted and unshifted groups tested equally on any given day, except for their prior history, these results were analyzed separately with one-way designs.

Baseline measurements did not differ between downshifted and unshifted groups for both test day conditions, *F*s < 1. Two main outcomes are observed in Fig 2. First, in groups tested after the first downshift session (32/17, 4/17), pain thresholds increase after 5 min, but they decreased after 300 min to match the values of unshifted controls. This was confirmed statistically. Relative to unshifted controls, downshifted animals exhibited hypoalgesia 5 min after session 17, *F*(1, 18) = 8.61, *p*<0.01, but not 300 min after that session, *F* < 1. Second, in groups tested after the second downshift trial, pain thresholds also increased in downshifted animals relative to unshifted controls after 5 min, *F*(1, 19) = 14.28, *p*<0.002, but, unexpectedly, this group difference remained significant even 5 hours after the end of the second downshift session, *F*(1, 19) = 13.58, *p*<0.003. No evidence of deviations from normality was detected on any of the significant effects shown in Fig 2 (statistics: < 0.23, *p*s>0.18).

The results reported above were not dependent on group differences in feeding motivation, as assessed in terms of body weight. The mean (±SEM) weights across sessions 1–19 were 239.6 (5.5), 240.0 (5.6), 232.4 (3.2), and 231.6 (4.1) g for Groups 32/17, 32/18, 4/17, and 4/18, respectively. A Contrast x von Frey analysis detected no effects, all *F*s < 1. A similar analysis on the mean weights during postshift sessions 17–19 also detected no effects, all *F*s < 1.

### Experiment 2

**Fig 3:**
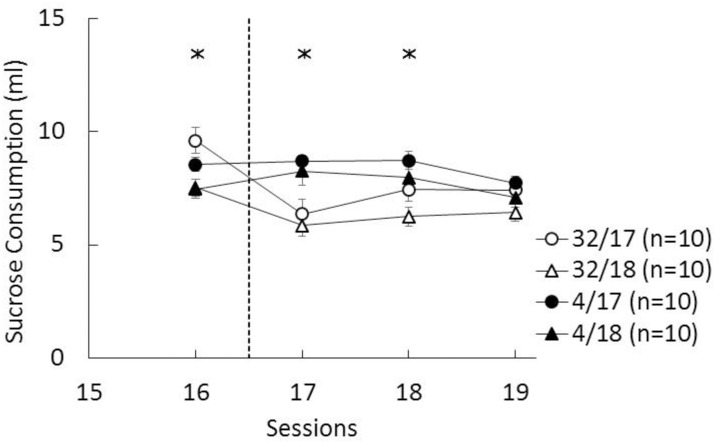
Mean (±SEM) sucrose consumption (ml) during the last preshift session (16) and postshift sessions (17–19). 32: animals exposed to a 32-to-4% sucrose downshift during postshift sessions. 4: animals exposed to an unshifted reward condition, receiving access to 4% sucrose throughout the experiment. The asterisks reflect significant differences between downshifted vs. unshifted groups (see text for details).

The results of the cSNC task were analyzed with Contrast (32% vs. 4% sucrose) x Hargreaves test (session 17 vs. 18) x Session analyses. Data from four individual sessions, one from each group, were lost due to technical difficulty; in these cases, the group average was substituted for the missing value. Preshift consummatory performance (sessions 1–16) showed that rats consumed significantly more 32% sucrose than 4% sucrose, as shown by significant effects for contrast, *F*(1, 36) = 84.33, *p*<0.001, and for the contrast by session interaction, *F*(15, 540) = 5.94, *p*<0.001. There was also a significant increase in consumption across preshift sessions, *F*(15,540) = 73.22, *p*<0.001. Other effects were not significant. Fig 3 shows the performance during the last preshift session, session 16; an analysis of just this session indicated a significant contrast effect, *F*(1, 36) = 11.85, *p*<0.002. Fig 3 also shows the results of the three postshift sessions, sessions 17–19. A similar analysis yielded a significant contrast by session interaction, *F*(2, 72) = 9.64, *p*<0.001. In addition, there were significant main effects for contrast and Hargreaves test, *F*s(1, 36) > 4.74, *p*s<0.04. The source of the contrast by session interaction was a significantly lower sucrose consumption of downshifted groups compared to unshifted controls on sessions 17 and 18, as indicated by LSD pairwise comparisons, *F*s(1, 36) > 11.61, *p*s>0.003. Across all sessions of training, there were no significant interactions involving the Hargreaves test factor, which indicated that group assignments were not biased. However, during postshift sessions, the groups tested for physical pain after session 17 consumed significantly more sucrose than the groups tested after session 18. Notice that since the triple interaction was nonsignificant, *F* < 1, the size of the cSNC effect was similar in both sets of downshifted-unshifted groups. There was no evidence of deviations from normality on sessions 16–19 (statistics: < 0.24, ps>0.12).

**Fig 4:**
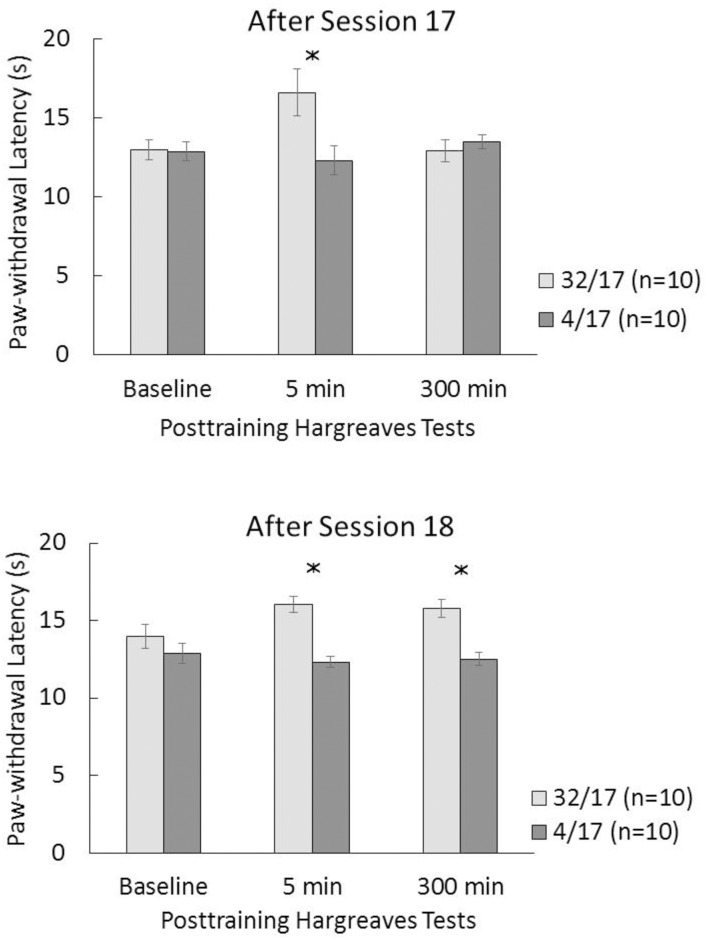
Paw-withdrawal latencies (s) recorded during the Hargreaves tests administered in baseline, and 5 or 300 min after sessions 17 (top) or 18 (bottom). 32: 32-to-4% sucrose downshift. 4: unshifted controls always exposed to 4% sucrose. In Group 32/17, 5 min, *n* = 8 due to loss of data; for all other measurements, *n* = 10. The asterisks reflect a significant difference between the corresponding downshifted vs. unshifted groups (see text for details).

Fig 4 shows the paw-withdrawal latency in the Hargreaves test for downshifted and unshifted groups tested after cSNC session 17 (top) or session 18 (bottom). As in Experiment 1, measurements were separated by different time intervals and, therefore, the results were analyzed separately for baseline, 5 min, and 300 min tests. Because of technical difficulties, data from two animals in Group 32/17, in the 5-min Hargreaves test, were lost, therefore leaving an *n* = 8 for this measure; however, those animals did produce data for baseline and 300-min tests, thus leaving an *n* = 10 for both of these measures. In addition, one of the two measurements in either the right or left paw was lost in 9 animals from Groups 32/17 and 4/17, in the 5-min test; in all these cases, the second measure taken from the corresponding paw was used in place of the average (the average of two measures was used when both were available).

No differences were observed in terms of baseline latencies either after session 17 or 18, *F*s(1, 18) < 1.17, *p*s>0.29. Pain sensitivity increased in downshifted groups relative to unshifted controls, as measured 5 min after session 17 or 18. Thus, latencies were longer in Group 32/17 than 4/17, *F*(1, 16) = 6.43, *p*<0.03, and in Group 32/18 than 4/18, *F*(1, 18) = 38.26, *p*<0.001. As in Experiment 1, the Hargreaves test yielded different effects at the 300-min interval depending on the session. After the first downshift event, latencies in Groups 32/17 and 4/17 had become nondifferential, *F* < 1. However, after the second downshift event, paw-withdrawal latency was still longer in Group 32/18 than in Group 4/18, *F*(1, 18) = 20.43, *p*<0.001. None of the significant effects shown in Fig 4 were based on samples that deviated from normality (statistics: < 0.26, *p*s>0.05).

The results reported above were not dependent on group differences in feeding motivation, as assessed in terms of body weight. The mean (±SEM) weights across sessions 1–18 (weights in session 19 were not taken) were 231.6 (±2.9), 232.3 (±3.3), 230.2 (±2.3), and 228 (±0.9) g for Groups 32/17, 32/18, 4/17, and 4/18, respectively. A Contrast x Hargreaves test analysis detected no effects, all *F*s < 1.33, ps>0.25. A similar analysis of the mean weights during postshift sessions 17–18 also detected no effects, all *F*s<1.

## Discussion

Three aspects of the present results merit discussion: (1) the relatively weak contrast effect observed in Experiment 1; (2) the issue of trial selectivity of the effects of reward devaluation on pain sensitivity; and (3) the postsession time course of these effects. We discuss below each of these in turn.

First, the cSNC effect observed in Experiment 1 was rather weak, compared to typical results in this task. Typically [5], the cSNC effect lasts between 1–5 sessions after the downshift, whereas in this case it lasted a single session and the difference between downshifted and unshifted groups was relatively small in absolute terms. The size of the cSNC effect was more conventional in Experiment 2, that is, downshifted groups were significantly suppressed during two postshift sessions, rather than one. Prior experience in a related task, iSNC, might have reduced the emotional impact of the 32-to-4% sucrose downshift in Experiment 1 (although this would not apply to the results of Experiment 2, in which animals had a similar prior experience). In Roman low-avoidance rats, which typically behave similarly to nonselected Wistars in SNC tasks [8,25], prior downshift experience in the iSNC task eliminated the cSNC effect in a subsequent phase [26]. To test for this possibility, the postshift consummatory behavior was analyzed again with the addition of a factor identifying whether the animal had prior downshift or unshift experience in the iSNC situation. The analysis yielded the same contrast by session significant interaction reported above, but none of the factors (main or interaction effects) involving prior experience was significant (Experiment 1: *F*s < 1.94, *p*s>0.17; Experiment 2: *F*s < 2.27, *p*s>0.14). In addition, we evaluated the potential effect of prior exposure to 2% ethanol during the iSNC experiment on the cSNC effect with Contrast x Ethanol x Session analyses for the preshift and postshift data. Again, no evidence of a main effect of ethanol or of any interaction with contrast was detected in these results (Experiment 1: *F*s < 3.08, *p*s>0.05; Experiment 2: *F*s < 1.86, *p*s>0.18). Thus, there was no evidence that prior downshift or ethanol experience had a measurable effect on the size of the cSNC effects observed in these experiments. There are individual differences in the extent to which rats respond to the downshift event in the cSNC situation [27,28] that are responsible for variability across experiments. What seems clear is that variations in the strength of the cSNC effects reported in these experiments did not prevent hypoalgesia from developing. Moreover, hypoalgesia was present after session 18 in Experiment 1, which had produced no evidence of contrast. Recent research in a different paradigm shows a similar decoupling of measures. Rats exhibit enhanced preference for ethanol and chlordiazepoxide over water immediately after a reward devaluation experience. Interestingly, preference for these anti-anxiety substances persists longer than the cSNC effect [29]. Results such as these suggest that even when animals appear to be behaviorally recovered from the negative effects of reward devaluation on consummatory behavior, other measures (including pain sensitivity in Experiment 1) indicate that they are still emotionally aroused.

Second, the issue of trial selectivity was raised by the results of an experiment similar to the present ones, except for using the hot plate as an assay of pain sensitivity [6]. In that experiment, hypoalgesia emerged after the second downshift session, but it was not detected after the first downshift session. A novel aspect of the present results is the finding that hypoalgesia was observed immediately after the first and second downshift sessions, thus providing no support for trial selectivity. These results suggest that trial selectivity may depend on the specific technique used to determine the animal’s sensitivity to physical pain. Moreover, the different techniques for assessing pain sensitivity used in these experiments suggest that the hypoalgesia observed after a frustrating experience is relatively independent to the peripheral pain receptors activated. Although both von Frey and Hargreaves tests activate potassium channels, mechanical stimuli stimulate acid-sensitive ion channels and ion channels of the degenerin family, whereas thermal stimuli activate transient receptor-potential channels [30]. The present experiments differed in some parameters relative to a previous experiment [6] involving the hot plate test, in which trial sensitivity was reported. For example, whereas rats had a single exposure to the hot plate, the present experiments involved baseline measurements before the key tests after reward devaluation were administered. In addition, stimulation of the plantar skin involves a wider set of receptors distributed over the four paws in the hot plate test than in the von Frey or Hargreaves test, in which stimulation is more localized and it stimulates receptors in only one paw [16]. The hot plate procedure typically involves exposure to a single temperature, whereas the von Frey test vary the strength of the nociceptive stimulus until a response is detected. It is unclear whether any of these procedural differences might account for trial sensitivity only with the hot plate test [6]. Notice, however, that the hypoalgesia observed 5 min after session 17 (first downshift session) was slightly weaker in both experiments than after session 18 (second downshift session)—although they were all significant (see Figs 2 and 4). A slight difference may be enough to produce evidence of trial selectivity of the type previously reported [6] if the technique for assessing peripheral pain is not sufficiently sensitive. Clearly, until a comparison of techniques for assessing pain sensitivity is undertaken, [31,32] their relative sensitivity for detecting interactions between physical and psychological pain will remain unknown. A similar situation was described for the relationship between reward devaluation and plasma corticosterone levels. Initially it was reported that corticosterone was elevated after the second downshift session, but not after the first downshift session [33]. However additional measurements with a different procedure detected differences after both sessions [34].

Third, studying the postsession course of hypoalgesia produced an unexpected outcome: Elevated pain thresholds 5 h after the end of the second downshift session. There are at least two possible explanations for this 300-min hypoalgesia effect, both dependent upon a repeated exposure to the downshift event (i.e., after two, rather than one, downshift sessions). It is possible that a second exposure to the downshifted reward caused a degree of emotional arousal that did not completely decay in the following 5 h. In fact, given that the level of hypoalgesia 5 min vs. 300 min after the second downshift session is virtually identical (see Figs 2 and 4), one would have to assume that there was no decay whatsoever in emotional activation. This is difficult to substantiate since by a variety of measures, the consequences of reward devaluation or omission, whether behavioral or physiological, seem to decay rather sharply in time (i.e., in the order of seconds to minutes, depending on the situation; [35,36]). Several sources of evidence support this conclusion: (1) Experiments involving surprising reward omissions result in invigorated performance when tested 2 or 4 s after the loss event, but the effect is gone after 20 s [36]; (2) Posttraining administration of corticosterone, which enhances the cSNC effect in subsequent sessions when administered immediately after session 11, has no measurable effect when administered 3 h after session 11 [21]; (3) Plasma corticosterone elevation was reported 10 and 20 min, but not 40 min after the second reward downshift session [33]; and (4) Previous exposure to an open field seems to have a bimodal effect on cSNC: It affects cSNC if admininstered 1 or 6 h before the downshift session, but not if administered 3 h or immediately before reward devaluation [37]. Overall, these results suggest a complex temporal dynamics of the behavioral and physiological consequences of reward loss.

The 300-min hypoalgesia effect could also depend on contextual reactivation of the devaluation event because of common features between the boxes where contrast and pain testing took place. Consummatory behavior in the cSNC task can come under contextual control [38], thus providing a possible mechanism. Some of the common elements involve the room in which both contrast and pain sensitivity tests were carried out, and the clear Plexiglas walls of both boxes (see Apparatus). This hypothesis assumes that a minimum number of two sessions of exposure to the reward devaluation event is necessary for the 300-min hypoalgesia effect to occur. If the effect depends on some minimum amount of exposure, then a more extensive duration for the first devaluation session should also lead to this 300-min hypoalgesia effect. For example, the effects of the GABAergic anxiolytic chlordiazepoxide on cSNC are not observed during the first devaluation session when it lasts 5 min, but they emerge later in that session if its duration is extended to 10 min [39]. Moreover, because the cSNC effect is transient, complete recovery from reward devaluation should eliminate the 300-min hypoalgesia effect. These hypotheses remain to be evaluated empirically.

The present results demonstrate the influence that psychological pain induced by reward devaluation can have over sensitivity to physical pain. Although the neurobiological mechanisms underlying such modulation are partially known [8], a more complete understanding awaits further study.
